# Structural and functional properties of the Kunitz-type and C-terminal domains of Amblyomin-X supporting its antitumor activity

**DOI:** 10.3389/fmolb.2023.1072751

**Published:** 2023-02-09

**Authors:** K. L. P. Morais, L. Ciccone, E. Stura, M. P. Alvarez-Flores, G. Mourier, M. Vanden Driessche, J. M. Sciani, A. Iqbal, S. P. Kalil, G. J. Pereira, R. Marques-Porto, P. Cunegundes, L. Juliano, D. Servent, A. M. Chudzinski-Tavassi

**Affiliations:** ^1^ Center of Excellence in New Target Discovery (CENTD), Butantan Institute, São Paulo, Brazil; ^2^ Laboratory of Development and Innovation, Butantan Institute, São Paulo, Brazil; ^3^ Department of Biochemistry, Federal University of São Paulo, São Paulo, Brazil; ^4^ Département Médicaments et Technologies pour la Santé (DMTS), Université Paris-Saclay, CEA SIMoS, Gif-sur-Yvette, France; ^5^ Department of Pharmacy, University of Pisa, Pisa, Italy; ^6^ Department of Pharmacology, Federal University of São Paulo, São Paulo, Brazil; ^7^ Department of Biophysics, Federal University of São Paulo, São Paulo, Brazil

**Keywords:** antitumor drug candidate, Amblyomin-X, TFPI-like, endocytosis, protein crystallization, high content imaging (HCI), proteasome inhibitor

## Abstract

Amblyomin-X is a Kunitz-type FXa inhibitor identified through the transcriptome analysis of the salivary gland from *Amblyomma sculptum* tick. This protein consists of two domains of equivalent size, triggers apoptosis in different tumor cell lines, and promotes regression of tumor growth, and reduction of metastasis. To study the structural properties and functional roles of the N-terminal (N-ter) and C-terminal (C-ter) domains of Amblyomin-X, we synthesized them by solid-phase peptide synthesis, solved the X-Ray crystallographic structure of the N-ter domain, confirming its Kunitz-type signature, and studied their biological properties. We show here that the C-ter domain is responsible for the uptake of Amblyomin-X by tumor cells and highlight the ability of this domain to deliver intracellular cargo by the strong enhancement of the intracellular detection of molecules with low cellular-uptake efficiency (p15) after their coupling with the C-ter domain. In contrast, the N-ter Kunitz domain of Amblyomin-X is not capable of crossing through the cell membrane but is associated with tumor cell cytotoxicity when it is microinjected into the cells or fused to TAT cell-penetrating peptide. Additionally, we identify the minimum length C-terminal domain named F2C able to enter in the SK-MEL-28 cells and induces dynein chains gene expression modulation, a molecular motor that plays a role in the uptake and intracellular trafficking of Amblyomin-X.

## Introduction

Coagulation abnormalities in cancer patients, dependent on tumor type and disease stage, lead to thrombosis (Falanga et al., 2013; Grandoni and Alberio, 2019). Alongside, activation of the coagulation cascade by cancer cells supports processes of tumor growth, metastasis, and angiogenesis (D'Asti and Rak, 2016; Schaffner et al., 2012). Its exact mechanism of action has not yet been completely elucidated, but a reasonable explanation for interconnecting hemostatic components and cancer biology is the production of the prime physiological initiator of coagulation, Tissue factor (TF) by tumor (Khorana et al., 2007; Ruf et al., 2016). Indeed, the antitumor effects of Tissue factor pathway inhibitor (TFPI) have been described in the last years, including essential hemostatic or non-hemostatic mechanisms (Amirkhosravi et al., 2007; Eisenreich et al., 2016; Graf and Ruf, 2018). Also, low levels of TFPI are associated with a bad prognosis in cancer cases (Stavik et al., 2011; Xu et al., 2013).

Mature TFPI protein consists of 3 tandem Kunitz inhibitor domains (K1, K2, K3). When TF is exposed on the cell surface, TFPI binds and inhibits TF-VIIa and Xa *via* K1 and K2 domains, respectively. The third domain has no inhibitory activity toward proteases but its C-terminal domain plays a role in cell membrane recognition, resulting in the internalization and degradation of this complex (Kato, 2002; Stavik et al., 2010; Stavik et al., 2013; Branco et al., 2016).

In this regard, Amblyomin-X is a homolog of Kunitz-type protein identified in the transcriptome of the salivary glands from the adult *Amblyomma sculptum* tick which shares about 40% similar to the K2 domain present in TFPI. As a recombinant protein, Amblyomin-X inhibits Factor Xa (FXa) activity *in vitro* and presents the anticoagulant property in animal models (Batista et al., 2010; Branco et al., 2016; Lobba et al., 2022). Notably, this molecule induced cytotoxicity in tumor cells (Chudzinski-Tavassi et al., 2010; Akagi et al., 2012; Ventura et al., 2013) and decreased tumor growth and metastasis *in vivo* (Chudzinski-Tavassi et al., 2010; Ventura et al., 2013; de Souza et al., 2016; Pavon et al., 2019; Lichtenstein et al., 2020) by non-hemostatic mechanism, including modulation of tumor immune microenvironment (Lichtenstein et al., 2020). After binding to phosphatidylserine exposed at the tumor cell surface, Amblyomin-X is internalized *via* a lipid microdomain and its intracellular trafficking is directed to the proteasome in human tumor cells (Pacheco et al., 2016). As a result of Amblyomin-X-dependent proteasome inhibition, proteolytic processing of NF-κB (a transcription factor that regulates cell survival) is blocked and a cell cycle arrest occurs (Chudzinski-Tavassi et al., 2010; Maria et al., 2013; Pacheco et al., 2014; Pacheco et al., 2016). In addition, Amblyomin-X induced several effects in human tumor cells, such as: aggresomes formation (Pacheco et al., 2014), autophagy inhibition (Pacheco et al., 2014), ER stress (Morais et al., 2016), mitochondrial dysfunction (de Souza et al., 2016; Morais et al., 2016), cytochrome-c release and PARP cleavage (Pacheco et al., 2014). All these effects taken together could culminate in apoptosis (Maria et al., 2013; Morais et al., 2016).

Of note, Amblyomin-X arises as a promising candidate to overcome one of the greatest challenges in the treatment of cancer, adverse effects, by differentiating between normal and cancer cells (Chudzinski-Tavassi et al., 2010; de Souza et al., 2016; Pacheco et al., 2016; Maria et al., 2019). Beyond antitumor affinity suggested previously (Boufleur et al., 2018; Graf and Ruf, 2018), preclinical studies have shown that Amblyomin-X does not cause any mortality and only a slight and reversible toxicity even at higher doses, thus establishing a safety profile for administration in mice (Maria et al., 2019). In agreement with this finding, Amblyomin-X treatment affected only tumor cells, making it an interesting candidate for anticancer therapy (Chudzinski-Tavassi et al., 2010; Morais et al., 2016; Pacheco et al., 2016; Schmidt et al., 2018). Even with much evidence about the effects and mechanism of action of Amblyomin-X in tumor cells, there is no evidence about how the N-terminal (N-ter) and C-terminal (C-ter) domains of this molecule are involved in these properties. Herein, we solved the crystallographic structure of the N-ter domain of Amblyomin-X, confirming its Kunitz-type signature. In addition, we investigated the biological relevance of both synthetic domains.

## Results

### Synthesis and refolding of the two Amblyomin-X domains

**FIGURE 1:**
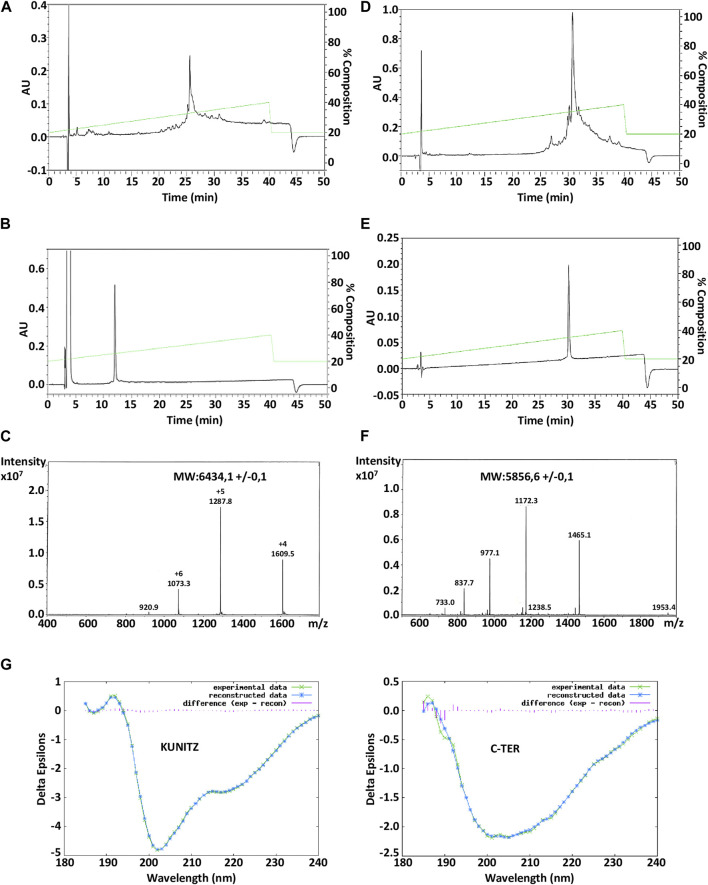
HPLC chromatograms of the crude Amblyomin-X-Nter (Kunitz domain) and Amblyomin-X-C-ter directly after cleavage **(A**, **D)** and after their refolding and/or purification **(B**, **E)**. ESI mass spectrum of the refolded Amblyomin-X-Nter **(C)** and AmblyominX-C-ter **(F)**. Circular dichroism analysis of Kunitz and C-ter domains of Amblyomin-X a. The spectra were measured at a peptide concentration of 20 μM **(G)**.

Amblyomin-X is a Kunitz-like FXa inhibitory protein of 106 amino acids and 7 cysteine residues. The synthesis of two truncated (synthetic) regions of Amblyomin-X, one called the Kunitz domain which corresponds to fragments (1–57) of Amblyomin-X contains the first 6 cysteine residues, the other the C-ter domain which corresponds to fragment 58-106 in which cysteine 67 has been mutated to the Serine residue, as well as the associated peptides, were performed by solid-phase peptide synthesis (SPPS). Due to the size of these peptides, we followed a strategy that we have successfully applied for the synthesis of long reticulated peptides (Mourier et al., 2016). The Fmoc SPPS strategy was combined with the use of a large excess of amino acids and HCTU/NMM as coupling reagents. To disrupt possible aggregation (data not shown) during synthesis, we incorporated commercial pseudoproline dipeptides, at position 20-21 (KT) in the Kunitz domain and at position 31-32 (LT) in the C-ter domain. For the synthesis of the p15-C-ter, an additional pseudoproline QT was added at position 14-15 (in linear numbering). The synthesis was successfully performed on 0.01 mmol scales in about 50 h and the polypeptides were easily isolated after HPLC purification with a yield of 13% (Kunitz), 9% (C-ter) (Figures 1A, B; Figures 1D, E) and characterized by ESI-MS (Table 1). Folding of the Kunitz domain and associated peptides were performed using a redox couple of reduced and oxidized glutathione associated with 0.5 M guanidine HCl. As shown in panels A and B of Figure 1, under these conditions, the Kunitz domain folded easily in 48 h as a single major component for a yield of 75% (Figure 1B). Their purity was confirmed by ESI-MS (Figures 1C, F; Table 1) and the CD spectrum performed in water shows the presence of a minimum at 205 nm and a shoulder between 215 and 225 nm, thus displaying a general shape common to other peptides associated with a Kunitz fold (Figure 1G). On the opposite, the CD spectrum of the C-ter domain displays a random structure (Figure 1G).

**TABLE 1:** Synthesis of Amblyomin-X domains and related peptides used in the study. Amino acid sequence and Molecular weight (average) by ESI mass spectrometry. *: amidation; C^#^: the 2 cysteines are connected by a disulfide bond.

Compounds	Amino acid sequence	MW average found	MW average calculated
Kunitz: 1-57 (57AA)	ANSKAVCNLPKAGDETCSNKTEIRWYYNGTACEAFIFKGCGGNDNNFDRVDDCQRLC-NH2	6434.1±0.1	6434.16
C-ter: 58-106 (49AA)	EEQTHFHFESPKLIFKVQDYWILNDIMKKNLTGISLKSEEEDADSGEID	5856.2±0.1	5856.4
TAT-Kunitz: 1-71 (71AA)	GRKKRRQRRRPPQANSKAVCNLPKLAGDETCSNKTEIRWYYNGTACEAFIFKGCGGNDNNFDRVDDCQRLC-NH2	8134.8±0.3	8135.17
p15-Cter: 1-61 (61AA)	C^#^WMSPRHLGT C^#^EEQTHFHFESPKLISFKVQDYWILNDIMKKNLTGISLKSEEEDADSGEID	7127.0±0.1	7126.9
TAT:1-13 (13AA)	GRKKRRQRRRPPQ	1718.01	1718.03
p15:1 -11(11AA)	C^#^WMSPRHLGTC^#^	1287.4	1287.5
F1C: 1-20 (20AA)	EEQTHFHFESPKLISFKVQD*	2445.6	2445.7
F2C: 16-35 (20AA)	FKVQDYWILNDIMKKNLTGI*	2438.8	2438.9
F3C: 31-50 (20AA)	NLTGISLKSEEEDADSGEID	2122.16	2122.18

Various chimeric peptides were synthesized to study the cytotoxic and cargo properties of Kunitz and C-ter domains of Amblyomin-X. The chromatograms and mass spectrum of TAT-Kunitz and p15-C-ter were described in Supplementary Figure S1.

### X-ray crystallography

The first needle crystals of Kunitz domain were obtained using the automatic robotic screening while the optimization was manually done. The diffraction limit of 1.60 Å was recorded for the best data set collected. The crystal structure of the Kunitz domain of Amblyomin-X belongs to the orthorhombic space group P2_1_2_1_2_1_ with two molecules in the asymmetric unit. The cell parameters are a = 26.54 Å, b = 46.09 Å, c = 76.17 Å and α = β = γ = 90° (Table 2).

**TABLE 2:** Crystallography of Kunitz domain of Amblyomin-X. Crystallographic data and refinement statistics.

Structure	Kunitz domain of Amblyomin-X
*PDB code*	**8AJ7**
*Data Collection Source*	SOLEIL Proxima2
Space group	P2_1_2_1_2_1_
*Unit Cell*	
*a*, *b*, *c* (Å)	26.54, 46.09, 76.17
α, *β*, γ (°)	90, 90, 90
Wavelength (Å)	0.980
Resolution (Å)	39.43–1.60
No. of reflections	119765
No. of unique reflections	12470
Completeness (%)	95.6 (95.7)
I/σ(I)	8.32 (1.85)
CC_1/2_	99.8 (0.75)
R_merge_ (%)	14.9 (115)
Multiplicity	8.2 (7.24)
*Refinement*	
Resolution (Å)	1.60
R_work_	0.256
R_free_	0.297
R.m.s. bond length deviation (Å)	0.008
R.m.s. bond angle deviation (°)	1.076
Ramachandra outliers (%)	0
favourable	95.37

**FIGURE 2:**
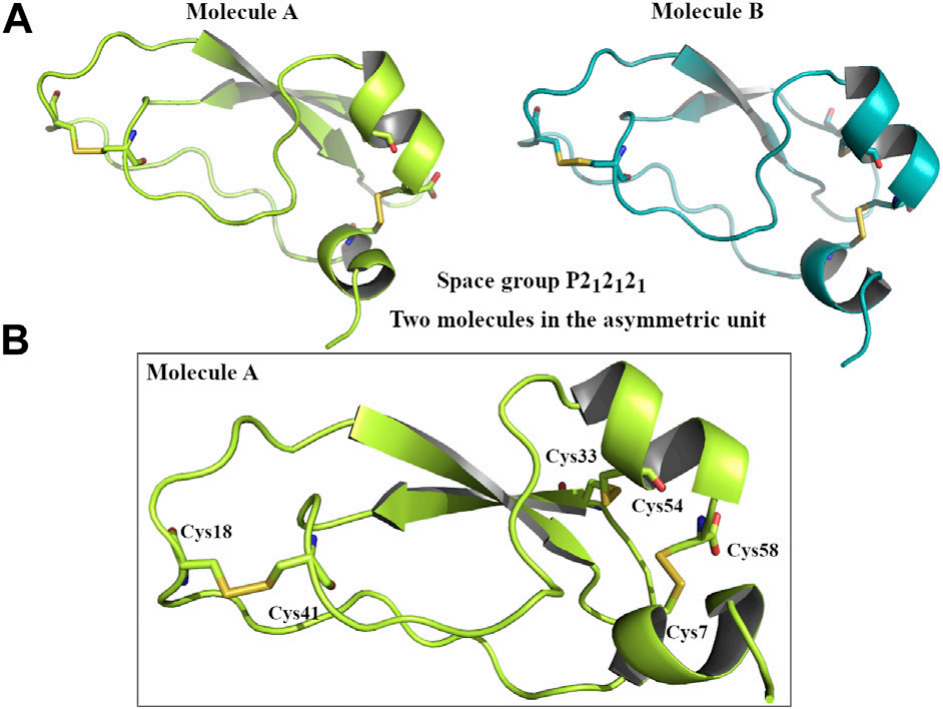
Graphical representation of Kunitz domain of Amblyomin-X. **(A)** Two molecules of Kunitz domain in the asymmetric unit. Molecule A coloured in lime and molecule B coloured in teal. **(B)** Focus of three disulphide bridges that characterizes the Kunitz domain of Amblyomin-X.

The X-ray analysis confirms that the first portion of Amblyomin-X (amino acids 1–58) possesses a typical Kunitz structure, with two α-helices and two β-sheets kept together with three disulfide bridges (Cys7-Cys58, Cys33-Cys54, Cys18-Cys41) (Figure 2).

Unfortunately, we did not succeed to obtain crystals neither for the C-ter domain nor for the complete Amblyomin-X sequence, excluding the resolution of their 3D structure.

### C-ter and Kunitz synthetic domains effects on tumor cells

**FIGURE 3:**
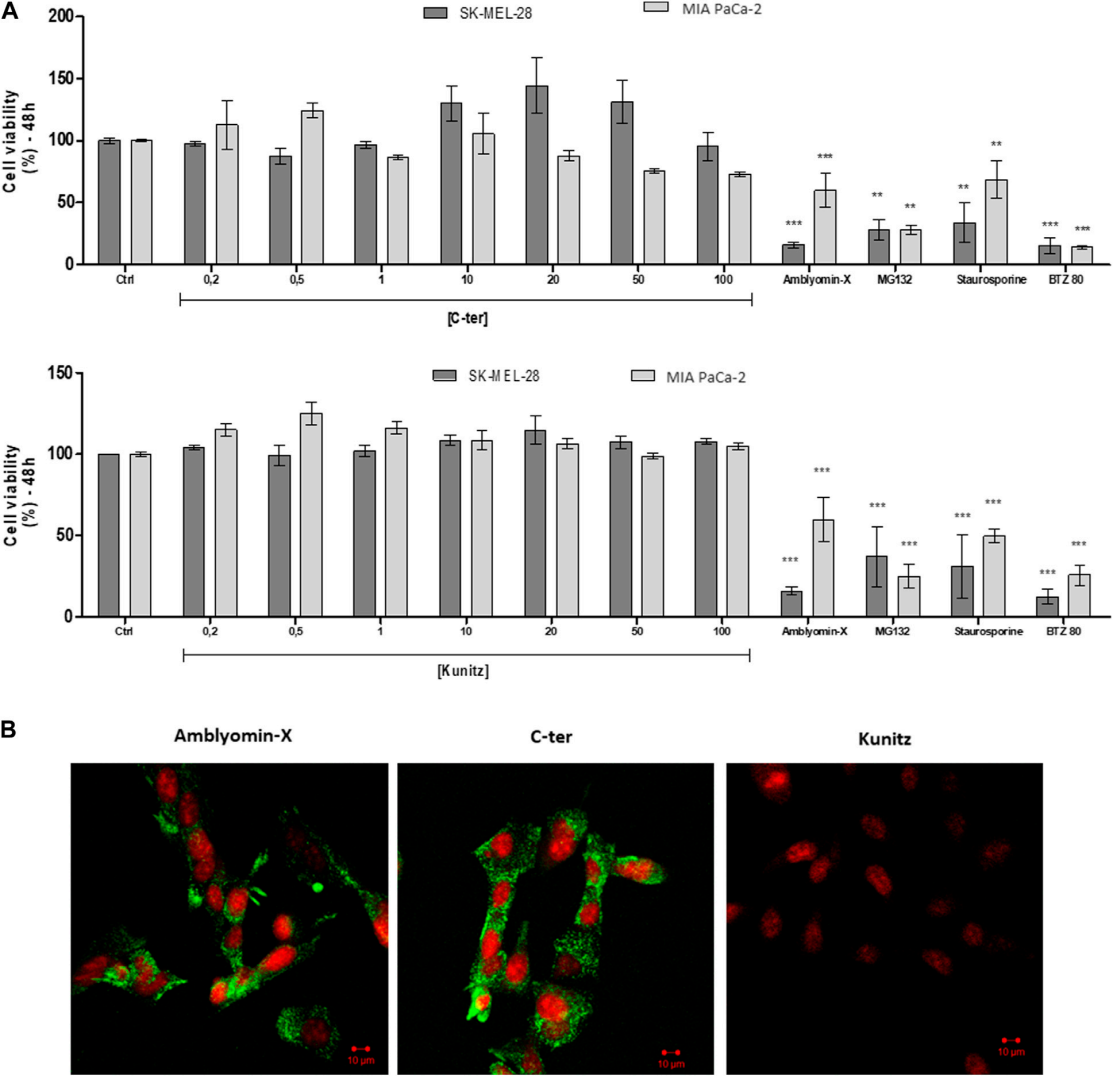
Amblyomin-X-induced cytotoxicity, but not of the isolated domains. **(A)** After 48 h, cell viability of tumor cells (SK-MEL-28 and MIA PaCa-2) was measured by MTT using increasing concentrations (µM) of C-ter and Kunitz. Positive controls: 20 µM full-Amblyomin-X, 5 µM staurosporine, 80 nM bortezomib (BTZ80) and 5 µM MG132. **(B)** Cells were incubated with 488-C-ter, 488-Kunitz or 488-Amblyomin-X (all at 20 µM), for 4 h. Green fluorescence was monitored with confocal laser scanning microscopy. Nuclei were stained with Syto59 (5 µM).

The effect of C-ter and Kunitz domains was investigated on the cell viability of human tumors cell lines already used in previous Amblyomin-X´s studies (Chudzinski-Tavassi et al., 2010; Pacheco et al., 2014; Morais et al., 2016; Pacheco et al., 2016; Schmidt et al., 2018), such as melanoma (SK-MEL-28) and pancreatic adenocarcinoma cells (MIA PaCa-2) by using MTT assays. After 48h, no alteration was observed. In contrast, Amblyomin-X and positive controls decreased the cell viability of both tumor cells (Figure 3A).

C-ter and Kunitz domains were labeled with cell-impermeable Alexa Fluor 488. After 4h, we observed that C-ter domain (20 µM) was uptaken by SK-MEL-28 (tumor cells) similarly to Amblyomin-X. On the opposite, the Kunitz domain (20 µM for 4 h) was not detected inside the cells (Figure 3B), suggesting that the C-ter domain should be responsible for the Amblyomin-X internalization.

### Caveolae and lysosome in the C-ter trafficking pathway

**FIGURE 4:**
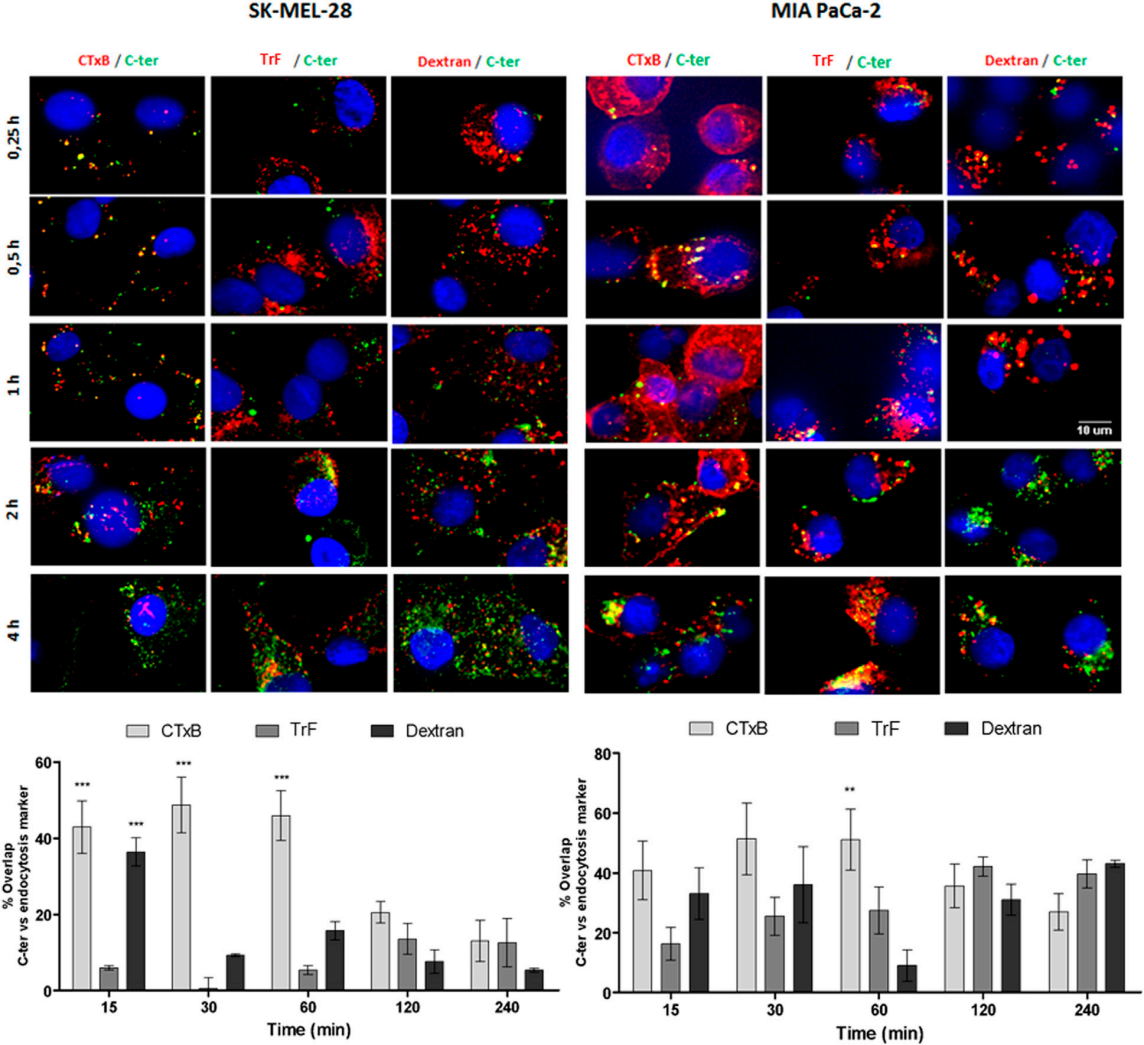
488-C-ter uptake started by lipid rafts. Tumor cell lines were treated with 488-C-ter at the indicated times; then Alexa Fluor 555 TrF (transferrin - 100 μg/ml), Alexa Fluor 555 cholera toxin subunit B (CTxB - 50 μg/ml) or Alexa Fluor 555 dextran (1 mg/ml) were added in the binding medium at 37 °C for 20 min. The nuclei were stained with Hoechst 33342. Next, automated images using ImageXpress Micro Confocal plate-scanning microscope (Molecular Device) at ×60 magnification at three wavelengths (for FITC, Texas Red and DAPI) were taken for nine fields per well. Overlap was analyzed using MetaXpress software (Molecular Devices, Sunnyvale, CA). Values are mean ± SD of three independent experiments (each one carried out using nine fields per well and in triplicate). *p ≤ 0.05; **p ≤ 0.01 and ***p ≤ 0.001 (comparing % of overlap between C-ter-488 and endocytosis markers at each time of treatment).

It has been suggested that Amblyomin-X uptake occurs *via* lipids raft because cholesterol removal promotes the reduction of Amblyomin-X inside the human tumor cells (Pacheco et al., 2016). Thereby, we investigated the intracellular route used by the C-ter domain by colocalization assay using Alexa Fluor 555-labeled dextran (Dextran), cholera toxin subunit B (CTxB) or transferrin (TrF). As seen in Figure 4, points of overlap were found between C-ter and all markers, but the highest ones were with CTxB, specifically in the shortest time of treatment, suggesting C-ter accumulation in caveolin positive structure.

**FIGURE 5:**
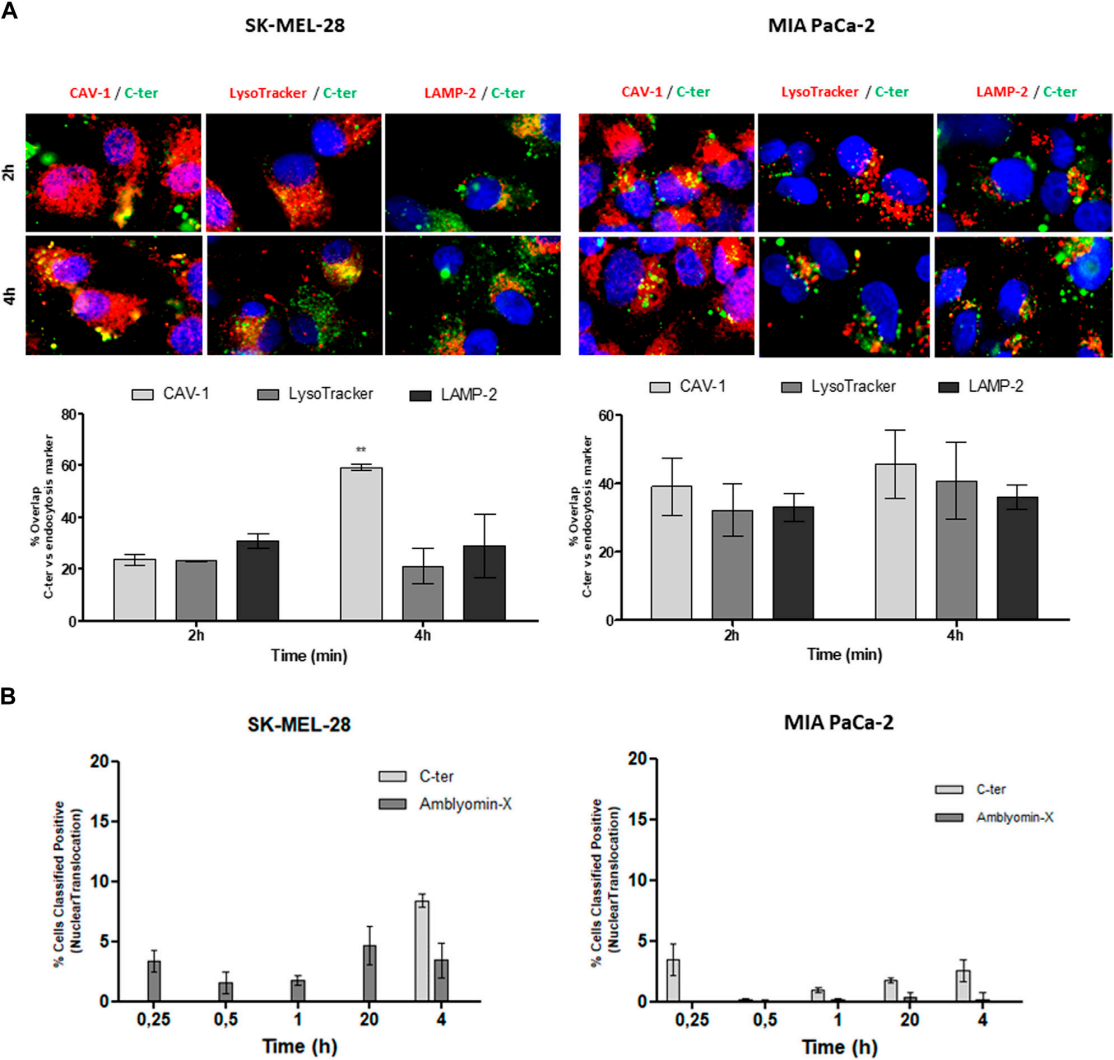
Participation of caveolin-mediated endocytosis and lysosomes in C-ter intracellular trafficking. **(A)** Tumor cell lines were treated with 488-C-ter at the indicated. After, cells were incubated with a primary antibody against caveolin-1 or LAMP-2, followed by a secondary antibody conjugated to Alexa Fluor 555. Also, after treatment, cells were then incubated with LysoTracker Red DND 99 (100 nM) in the binding medium at 37°C for 20 min. Then, green and red fluorescences were monitored by a Molecular Devices ImageXpress Micro Confocal plate-scanning microscope. Nuclei stained with Hoechst 33342. The percentage of cells positive for nuclear translocation **(B)** and overlap between C-ter and intracellular markers were analyzed using MetaXpress software (Molecular Devices, Sunnyvale, CA). Values are mean ± SD of three independent experiments (each one carried out using nine fields per well and in triplicate). *p ≤ 0.05; **p ≤ 0.01 and ***p ≤ 0.001 (comparing % of overlap between 488-C-ter-488 and intracellular markers at each time of treatment). % Classified Positive Nuclear translocation (the number of cells classified as positive for translocation divided by the total cell count, times 100) was calculated by using Translocation Application Module of MetaXpress software (Molecular Devices, Sunnyvale, CA).

In agreement, co-localization with cav-1 was observed (Figure 5A) mainly after 4 h of treatment. Also, C-ter was found in the co-localization with late endosomal markers, LysoTracker and LAMP-2, especially in MIA PaCa-2. In addition, we quantified C-ter and Amblyomin-X uptake and observed that C-ter accumulates inside the cells faster than Amblyomin-X (Table 3). Few cells were positive for nuclear translocation (Figure 5B).

**TABLE 3:** C-ter acumulation inside the cells faster than Amblyomin-X. Tumor cells were treated with Alexa-Fluor 488-labeled molecules (20 µM) at indicated times. Fluorescence were monitored by a Molecular Devices ImageXpress Micro Confocal plate-scanning microscope (Molecular Devices, Sunnyvale, CA). Vesicles counts were analyzed using MetaXpress software (Molecular Devices, CA) with Transfluor Application Module.

Cell type	Time (h)	C-ter	Amblyomin-X
SK-MEL-28	0.25	30.84 ± 17.2	15.13 ± 2.71
	0.5	35.56 ± 21.09	29.89 ± 12.27
	1	62.17 ± 11.20	35.23 ± 17.32
	2	558.48 ± 189.00	139.57 ± 71.44
	4	1210.74 ± 379.14	988.76 ± 409.20
Mia PaCa-2	0.25	4.36 ± 2.03	0.38 ± 0.25
	0.5	17.50 ± 24.63	2.77 ± 1.96
	1	10.59 ± 7.15	7.42 ± 0.81
	2	171.11 ± 248.57	76.69 ± 20.72
	4	802.63 ± 353.12	1672.38 ± 395.60

### Crossing the cell membrane is necessary for the Kunitz domain to induce cytotoxicity

**FIGURE 6:**
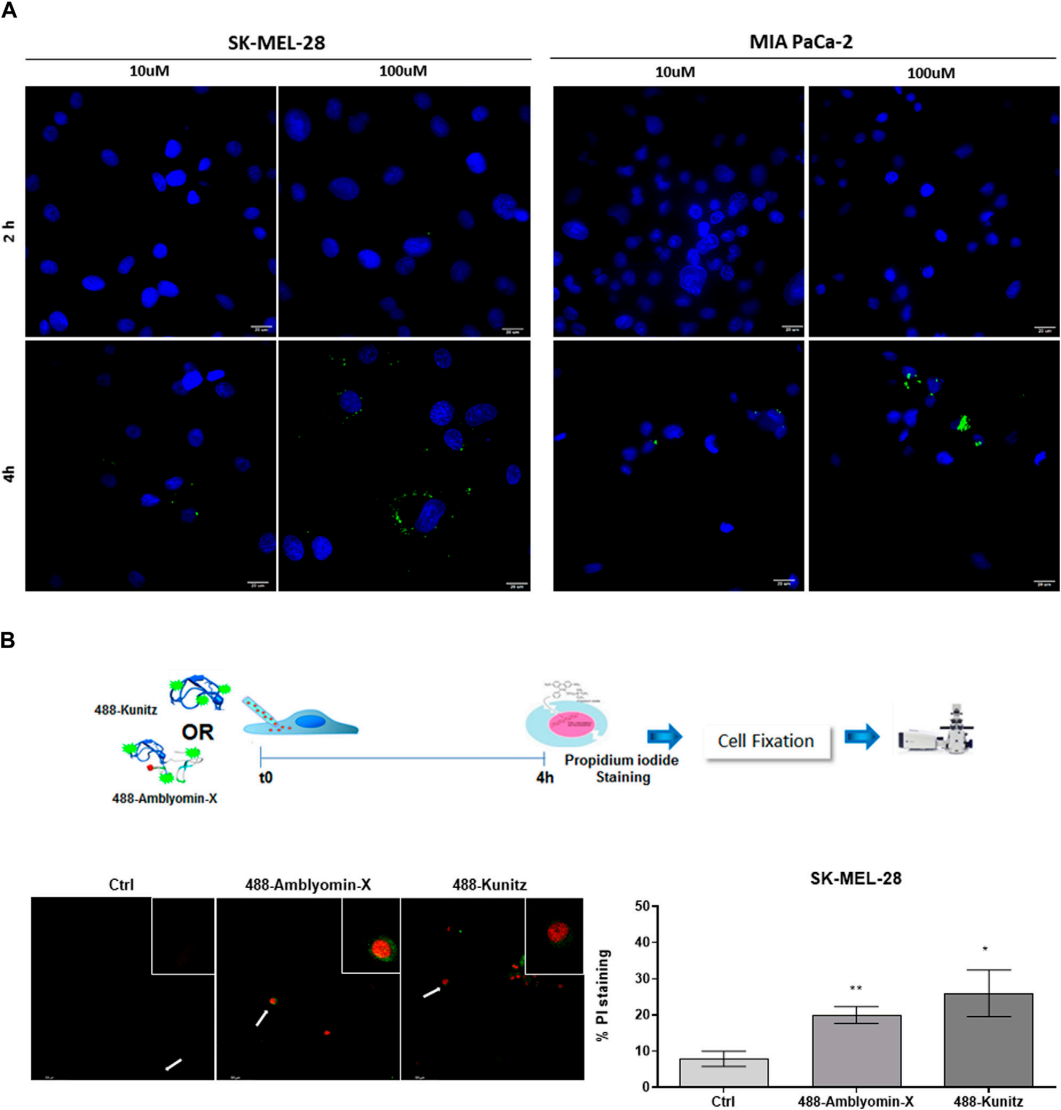
Kunitz is slightly uptake by tumor. **(A)** Cells were incubated with 488-Kunitz (10 μM and 100 µM) for 2 and 4 h. Green fluorescence was monitored by a Molecular Devices ImageXpress Micro Confocal plate-scanning microscope. Nuclei stained with Hoechst 333422. Kunitz vesicles were analyzed using MetaXpress software (Molecular Devices, Sunnyvale, CA). **(B)** Cells were microinjected with 488-Kunitz or 488-Amblyomin-X, both at 20 μM, and incubated for 4h, when cell death was scored, based upon green fluorescence and PI staining.

We investigated the highest concentrations of the Kunitz domain (100 µM) in uptake studies. After 4 h of treatment, the number of Kunitz vesicles was ten-fold less than complete Amblyomin-X (Figure 6A; Table 4).

**TABLE 4:** Cellular-uptake efficiency low of Kunitz molecule. Tumor cells were treated with Alexa-Fluor 488-labeled molecules at indicated times. Fluorescence were monitored by a Molecular Devices ImageXpress Micro Confocal plate-scanning microscope (Molecular Devices, Sunnyvale, CA). Vesicles counts were analyzed using MetaXpress software (Molecular Devices, CA) with Transfluor Application Module.

Cell type	Time (h)	Kunitz (10 µM)	Kunitz (100 µM)	Amblyomin-X (20 µM)
SK-MEL-28	2	0.26 ± 0.024	4.14 ± 3.76	139.57 ± 71.44
	4	1.12 ± 0.11	37.64 ± 0.16	988.76 ± 409.20
MIA PaCa-2	2	0.8 ± 0.28	13 ± 5.93	76.69 ± 20.72
	4	1.86 ± 1.31	98 ± 49.73	1672.38 ± 395.60

Also, after full-Amblyomin-X or Kunitz microinjection (both 20 µM), propidium iodide (PI) staining was similarly detected in SK-MEL-28, 20 and 26%, respectively (Figure 6B), measuring a decrease in cell viability. No microinjection cells were used as control. This result indicates that the Kunitz domain could be responsible for cytotoxicity and its internalization is crucial for this effect.

### Cargo-delivery ability of C-ter domain and Kunitz domains uptake by TAT-fusion

**FIGURE 7:**
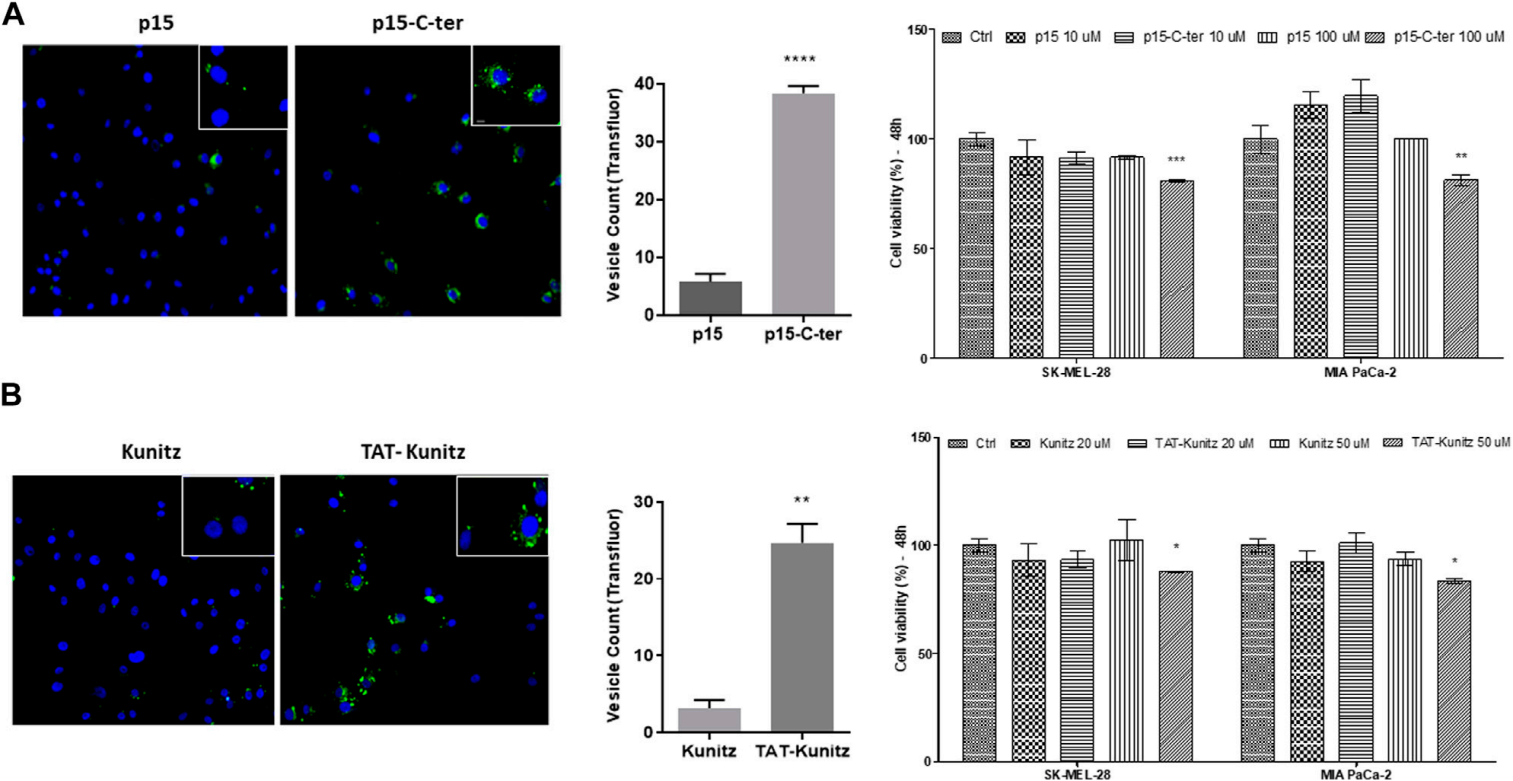
Cargo-delivery ability of C-ter and intracellular detection of TAT-Kunitz. **(A)** SK-MEL-28 cells were treated with p15 or P15-C-ter both Alexa Fluor 488-labeled for evaluation of its uptake and cell viability was investigated by MTT assay. **(B)** Kunitz uptake and cytotoxicity by using TAT CPP delivery tool. In all approaches, nuclei were stained with Hoechst 333422 and green fluorescence was monitored by a Molecular Devices ImageXpress Micro Confocal plate-scanning microscope. Vesicles were defined using MetaXpress software (Molecular Devices, Sunnyvale, CA) with Transfluor Application Module. Values are mean ± SD of three independent experiments (each one carried out using nine fields per well and in triplicate). *p ≤ 0.05; **p ≤ 0.01 and ***p ≤ 0.001 (comparing CPP-conjugated molecule vs. non-conjugated).

P15 is a protein kinase CK2 inhibitor that shows proapoptotic and antiproliferative activities *in vitro* affecting transformed cells of different origin when conjugated with the cell-penetrating peptide TAT (Perea et al., 2004; Cozza et al., 2013). After 4h, p15-C-ter (10 μM) was eight-fold more internalized than the non-conjugated molecule, highlighting the cargo-delivery property of the C-ter domain (Figure 7A). Also, p15-C-ter (100 μM) decreased cell viability in both tumor cells (SK-MEL-28 and MIA PaCa-2), suggesting that the amount of molecule needed to trigger a cytotoxic effect was reached. Furthermore, a large enhancement of Kunitz uptake was observed after its fusion with the TAT peptide associated with a significant cytotoxicity activity (Figure 7B).

Identification the minimum length C-terminal domain able to enter in tumor cells and modulate dynein chains. In order to determine the region of the C-ter domain involved in the Amblyomin-X internalization process, three overlapping fragments were synthesized (F1C, F2C, F3C, Table 1), conjugated to fluorescein isothiocyanate (FITC) and assessed for their ability to internalize in SK-MEL-28 cells.

**FIGURE 8:**
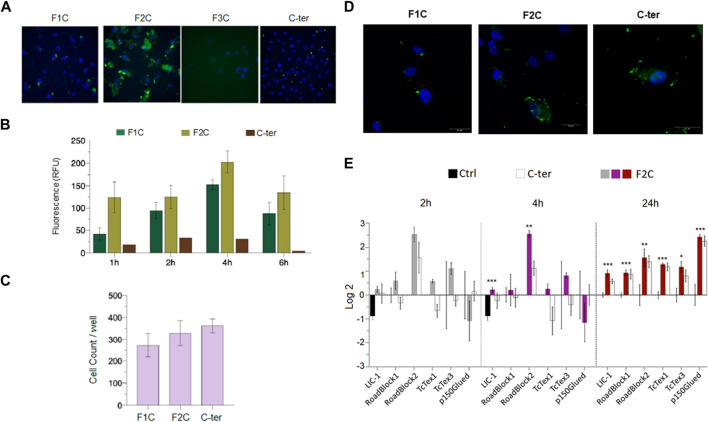
F2C, a C-ter derived fragment uptaken by tumor cells and its action on dynein chains. SK-MEL-28 cells were treated with fluorescent peptides F1C, F2C, F3C and C-ter (all at 10 µM) for indicated times. After 6h, uptake was evaluated. **(A)** Representative figures after 6 h of treatment. **(B)** Quantification of relative fluorescence units (RFU). **(C)** Cells counts per well. **(D)** Confocal analysis. **(E)** Cells were treated with F2C or C-ter (5 µM) at the indicated times and levels of gene expression dynein chains were evaluated by real-time PCR. Green fluorescence was monitored by a Molecular Devices ImageXpress Micro Confocal plate-scanning microscope or by Confocal microscope TCS SP8 (Leica Microsystems, GmbH Mannheim, DE). RFU and cells count were defined using MetaXpress software (Molecular Devices, Sunnyvale, CA). Values are mean ± SD of three independent experiments. *p ≤ 0.05; **p ≤ 0.01 and ***p ≤ 0.001.

As expected, C-ter domain (5 or 10 µM for 6 h) was uptaken by SK-MEL-28 while of the three fragments only F1C and F2C were detected in the cell (Figures 8A, B). Interestingly, higher values of relative fluorescence units (RFU) were found for F2C even in short times of treatment, indicating efficient uptaken and fast accumulation (Figure 8A). No alterations in cells counts were observed after all treatment investigated, suggesting neither the C-ter nor its fragments affect cell viability (Figure 8C). Collaborating with this data, F2C modulate gene expression of all dynein chains evaluated after 24 h (Figure 8E).

## Discussion

Hemostatic components and cancer biology are interconnected in multiple ways. In this regard, endogenous regulator of the coagulation cascade, TFPI, plays an important role in stopping coagulation and cancer progression by inhibiting the extrinsic tenase complex formed by the TF, the active factor VII (FVIIa), and the active factor X (FXa) on the tumor cells surface, and the FXa alone through its first (K1) and second (K2) Kunitz-domain, respectively (Crawley and Lane, 2008). While K1 and K2 domains form a tight complex with coagulation factors and inhibit their proteolytic activity (Girard et al., 1989), the C-terminal portion of TFPI interacts with low density lipoprotein (LDL) receptor family (LRP-1 and VLDL) (Warshawsky et al., 1994), which bridges the FVII/TF complex to LRP1. This results in the internalization of the TF/FVIIa/FXa complex and the down-regulation of TF-mediated coagulant activity (Warshawsky et al., 1994).

In addition, TFPI inhibits the intracellular activity of FXa and the TF/FVIIa/FXa complex by inhibiting the activation of the protease activated receptor 2 (PAR-2) (Camerer et al., 2000), which in turn induces downstream signaling leading to the production of pro-angiogenic factors, inflammatory cytokines and growth factors, all them essential to support tumor cell migration and metastasis (Unruh and Horbinski, 2020). In this context, several studies have demonstrated that Amblyomin-X is functionally related to TFPI and TFPI-like inhibitors since it is able to inhibit the FX activation by FVIIa/TF tenase complex in a concentration-dependent manner (Batista et al., 2010; Morais et al., 2016). Along the same line, Amblyomin-X is a promising candidate for cancer treatment showing anticoagulant, antitumor and antimetastatic activities as well as tumor affinity and cytotoxicity preferentially in tumor cells (Batista et al., 2010; Chudzinski-Tavassi et al., 2010; Pacheco et al., 2014; Branco et al., 2016; de Souza et al., 2016; Morais et al., 2016; Pacheco et al., 2016; Schmidt et al., 2018). Although the N-ter domain of Amblyomin-X has a high similarity with K2 domain of the TFPI, the respective functional role of the N-ter and C-ter domains of Amblyomin-X were not yet explored.

In the present study, N-ter and C-ter domains of Amblyomin-X were chemically synthesized by solid-phase synthesis. The N-ter was confirmed as a typical Kunitz structure showing two α-helices and two β-sheets kept together with three disulfide bridges resulting in the cyclic disulfide-rich peptide (Figure 2), according to the previous published model (Batista et al., 2010), a feature that improved molecules resistance to proteolytic degradation, and it is used as a strategy during drug design (Craik, 2006; Wang and Craik, 2018; Chan et al., 2021). The crystal structure of the Kunitz domain of Amblyomin-X grew in the orthorhombic space group P2_1_2_1_2_1_ with two molecules in the asymmetric unit that are almost identical (rmsd = 0.08 Å). The superimposition between the crystal structures of Kunitz domain of Amblyomin-X and TFPI-K2 (pdb 1TFX) showed the high similarity of the two Kunitz domains (rmsd = 1.2 Å). The main difference is remarkable around the TFPI-K2 binding loop (Pro11-Thr19) where the loop of Kunitz domain of Amblyomin-X (Ala13-Glu23) is longer than those of the human TFPI-k2 (Supplementary Figure S2). It has been reported that, the loop of TFPI-K2 comprised by Pro11-Thr19 is important for the interaction with the FXa (Burgering et al., 1997). It is interesting to highlight that the side chain residues exposed by the Kunitz domain of Amblyomin-X are different in this region respect those of TFPI-K2 (Supplementary Figure S2). The diversity between the two loops of these Kunitz domains could suggest a different interaction of Kunitz domain of Amblyomin-X with the FXa. This consideration is in agreement with the evidence that Kunitz domain of Amblyomin-X showed lower inhibitory activity against FXa than TFPI-K2 (Supplementary Figure S4). Regarding the full length of Amblyomin-X we did not obtained the crystals, probably because of the intrinsic disorder of the C-ter domain, in particular the last part of its sequence (SEEEDADSGEID) as suggested by the flDnn server (http://biomine.cs.vcu.edu/servers/flDPnn/) (Supplementary Figure S3).

In addition, Kunitz cytotoxicity and uptake by tumor cells were observed only if this domain is conjugated to a cell-penetrating peptide (TAT) or directly microinjected in the cytoplasm. Initially, these results indicate that Kunitz-type domain is associated with full-Amblyomin-X cytotoxicity. However, Kunitz’s cytotoxicity was achieved with higher doses than observed for Amblyomin-X and these approaches could not mimic biological conditions, i.e., TAT could lead to activation of different types of endocytosis as well as direct translocation *via* the plasma membrane or cytoplasmic proteases inhibition by microinjection.

Unlike Kunitz, C-ter domain was responsible for Amblyomin-X uptake since fluorochrome-conjugated C-ter domain was detected inside of tumor cells while no cytotoxicity was observed in these treated cells (Figures 3, 4) and its accumulation inside the cells was faster than Amblyomin-X (Table 3). Similarly, to full-Amblyomin-X, C-ter were intracellular trafficking undergo caveolin-positive structure evidenced by preferentially co-localization with CTxB and cav-1 in SK-MEL-28for this domain. Distinctly, in MIA PaCa-2 cells no pathway seems to be predominant. Indeed, MIA PaCa-2 cells are less sensitive to Amblyomin-X cytotoxicity and there are some differences regarding intracellular events, for example, in the modulation of dynein, a molecular motor that plays a role in the uptake and intracellular trafficking of Amblyomin-X (Pacheco et al., 2014; Pacheco et al., 2016). In both tumor cells, nucleus is not the main fate of C-ter (Figure 5B).

Reinforcing the role of C-ter in the uptake process of Amblyomin-X, its cargo-delivery ability was demonstrated by enhancement uptake and biological activity of the p15 peptide (Figures 7A, B). However, C-ter has no conventional features of CPPs, such as sequence, chemical charge, binding type, and general physicochemical properties. In general, CPPs are typically not exceeding 30 residues in length and often carry a positive charge, which facilitates electrostatic interactions with negatively charged cell-surface (Benavent Acero et al., 2014; Kristensen et al., 2016). In contrast, C-ter has 50 residues and a negative formal charge. Even with extrapolating comparisons, for instance, evaluating CPPs longer than 30 residues, such as VP22, IR9, Protamine, LL-37 and maurocalcine we couldn’t find a pattern, because there is a mixture of different endocytic mechanisms including clathrin-mediated, non-clathrin-mediated and processes such as micropinocytosis (Tisseyre et al., 2014; Wierzbicki et al., 2014; Kristensen et al., 2016; Ponnappan and Chugh, 2017; Yamashita et al., 2017). Thereby, cell delivery role was investigated through assays using different synthetic C-ter-fragments. Among tested peptides, F2C is the only with positive formal charge (pI: 8.43), a conventional features of CPPs. In agreement, C-ter and F2C modulated gene expression of dynein chains unlike other fragments and TAT-Kunitz (data not shown). These dynein chains modulated by C-ter are related to interactions with binding partners that target and regulate dynein motor activities and cargo diversity and different intracellular transport (Tai et al., 1998; Cheung et al., 2004; Mische et al., 2008; Stuchell-Brereton et al., 2011). Interestingly, alignment of multiple protein sequences analysis shown similarity between C-ter and other proteins, which reasonable explanation for different intracellular pathways triggers by Amblyomin-X, such as dynein recruitment (Supplementary Figure S5B). Notably, peptides similar to the synthetic compounds studied here were found in the lysate generated from tumor cells treated with Amblyomin-X (Supplementary Figure S5A).

Overall, the data reported here support C-ter domain is responsible for Amblyomin-X uptake by tumor cells as well as regulation of it intracellular destination. As Kunitz has less biological activity than the whole protein, C-ter seem to be required for conformational structure or resistance to further degradation, which could be associated.

Random structure of C-ter, indicating high flexibility, which could influence short-range protein-protein interactions. Even as Kunitz-type domain (cysteine conserved) for serine protease inhibition, this feature must be important for full-Amblyomin-X activity. Kunitz-type domain of Amblyomin-X is associated with its tumor cells cytotoxicity. Thus, these results are relevant to understanding the Amblyomin-X mechanism of action, which is a promising molecular entity to treat malignant tumors and opening frontiers for future experimentation, for example, using C-ter-derived peptides or Kunitz domain as a cyclic disulfide-rich scaffold.

## Methods

### Materials

Fmoc-amino acids, Fmoc-pseudoproline dipeptides, and 2-(6-chloro-1H-benzotriazole-1-yl)-1, 1, 3, 3, -tetra-methylaminium hexafluorophosphate (HCTU) were obtained from Novabiochem (Darmstadt, Germany). The resins and all the peptide synthesis grade reagents (N-methylpyrrolidone (NMP), Nmethylmorpholine (NMM), dichloromethane, piperidine, trifluoroacetic acid (TFA), anisole, thioanisole, triisopropylsilane) and 5-carboxyfluorescein were purchased from Sigma (Saint-Quentin Fallavier, France).

### Peptide synthesis

*Two truncated (synthetic) regions of Amblyomin-X, Kunitz and C-ter domain, and related peptides*: the syntheses were performed on a Protein Technologies, Inc., prelude synthesizer at a 12.5 µmoles scale using a 10-fold excess of Fmoc-amino acid relative to the resin loading, respectively an amide chemmatrix resin (0.33 mmol/g) or a Fmoc-Asp(tBu)-wang-LLresin (0.33 mmol/g). Fmoc-protected amino acids were used with the following sidechain protections: tert-butyl ester (Glu and Asp), tert-butyl ether (Ser and Tyr), trityl (His, Asn, and Gln), tertbutoxycarbonyl (Lys), and 2, 2, 5, 7, 8-pentamethyl-chromane-6- sulfonyl (Arg). Amino acids were coupled twice for 10 min using 1:1:2 amino acid/HCTU/NMM in NMP, except positions 25, 26, 27, 28 in the Kunitz domain peptide where four coupling of 10 min were applied. A pseudo proline dipeptide (KT) was used at positions 20-21 in the Kunitz domain, a pseudo proline (LT) at position 31-32 in the C-ter domain and pseudoproline dipeptides QT and LT were used at position 14-15 and 43-44 in the p15-C-ter (in linear numbering). All of these dipeptides were coupled twice for 10 min. After incorporation of each residue, the resin was acetylated for 5 min using a 50-fold excess of a mixture of acetic anhydride and NMM in NMP. Fmoc deprotection was performed twice for 3 min using 20% piperidine in NMP, and 30 s NMP top washes were performed between deprotection and coupling steps and after acetylation.

Finally, all the peptidyl-resins were treated with a mixture of Trifluoroacetic acid (TFA)/thioanisole/anisole/Trisodium phosphate(TPS)/water (82:5:5:2.5:5) for 2 h. The crude peptides wereobtained after precipitation and washes in cold ethyl ether followed by dissolution in 10% acetic acid and lyophilized. The different peptides were purified by reverse phase HPLC using an X-Bridge BHE C18-300-5 semi-preparative column (250 × 10 mm) or analytical column (250 × 4.6 mm) (Waters, United States). The flow rate was 4 ml/min^−1^ and we used a gradient of 20–40% or 10–40% solvent B into A in 60 or 40 min; solvent A (H_2_O/TFA 0.1%) and solvent B (Acetonitrile/TFA 0.1%). The purity of each peptide was checked by mass spectrometry using ESI-MS (Bruker, Germany). Amino acid sequence of all compounds are shown in Table 1.

### Refolding and disulfide formation

*Kunitz domain*: Refolding were performed at 4°C in Tris 0.1 M, EDTA 1 mM buffer pH 8, containing 0.5 M of guanidine-HCl (Gdn-HCl), 1 mM reduced glutathione (GSH) and 1 mM oxidized glutathione (GSSG). Peptide concentration was 10 µM. After 48h, the reaction was stopped by acidification and purified by HPLC (column BEH300 XBridge C18, 5 µm. 4.6 × 250 mm).

*P15 peptides*: Disulfide formation was done at room temperature (r.t.) and in a flask open to the atmosphere in 0.1 M ammonium hydrogen carbonate solution pH 7.8 at a concentration of 0.1 mg/ml until the reaction was complete (HPLC monitoring) and the purity of each peptide was checked by mass spectrometry using ESI-MS (Bruker, Germany).

### Circular dichroism

CD spectra were recorded on a JASCO J-810 spectropolarimeter equipped with a thermoelectric sample temperature controller (Peltiersystem). C-ter and Kunitz domains samples (20 μM) were diluted in 20 mM phosphate buffer (PBS), pH 7.4, or water using a total volume of 550 μL. Milli-Q water and buffer without protein were used as blank. The scans were collected from 190 to 260 nm at r.t. using a pathlength quartz cell (Helma) of 1.0 mm (200 µL). The data were corrected and adjusted to the input buffer and the mean molar residual ellipticity was calculated based on a molecular mass of each compound. The estimation of secondary structure was performed using CDNN CD spectra deconvolution software (Bohm et al., 1992) and DicroWeb program (Deleage and Geourjon, 1993).

### Crystallization and structure determination of Kunitz domain of Amblyomin-X

The synthesized Kunitz domain (amino acid 1–58) of Amblyomin-X was used for crystallization experiments. Protein crystallization robot mosquito^®^ SPT Labtech was used for preliminary crystallization trials and several conditions were tested. Microplate for 96-wells was filled by 200 μL of reservoir solution, and the drops were automatically dispensed by the mosquito^®^ robot. 150 nL of Kunitz solution (10 mg/ml) was equilibrated against 150 nL of precipitant. Condition PEG_2D (45% polyethylene glycol (PEG) 600, 0.1 HEPES pH 7.5) of Stura Footprint Screen (Molecular Dimensions Ltd., United Kingdom) gave first needles crystals.

The crystals optimization was manually made using sitting drop vapor diffusion CrysChem plates. The plates were equilibrated in a cooled incubator at 20°C. Starting from the condition identified by the mosquito^®^ robot, several crystallization conditions were tested following the strategy of reverse screening (Stura et al., 1994).

To induce nucleation and encourage crystal growth, a booster solution, consisting of 5 M NaCl and or 5 M NaCl with 0.2 M acetic acid, was added at the reservoir and the drop immediately streak seeded (Ciccone et al., 2015a; Ciccone et al., 2016). Seeds were picked up by the drop made by mosquito^®^ robot. The crystal analyzed in this study was grown from 0.6 μL protein solution (9 mg/ml) and 1.4 μL precipitant from the reservoir (500 μL). The reservoir solution was prepared mixing together two standard working solutions: 50% PEG_2C (36% PEG 600, 0.1 HEPES pH 7.5) and 50% (36% PEG 2000, 0.1 sodium cacodylate pH 6.5) of Stura Footprint Screen.

For data collection, the crystals were cryoprotected by quick immersion into various cryoprotectant solutions (Ciccone et al., 2015b) before flash freezing in liquid nitrogen. The data sets of several complexes were collected at synchrotron facility SOLEIL at microfocus beamline Proxima2a, Gif-sur-Yvette, France using both standard rotation and helical scan method (Polsinelli et al., 2017).

About 30 samples were tested and the best crystal diffracted to 1.60 Å. X-ray diffraction data were collected from a single crystal at 100 K on DECTRIS EIGER X 9M detector. The crystal belongs to the space group P2_1_2_1_2_1_ with unit cell parameters of a = 26.54 Å, b = 46.09 Å and c = 76.17 Å. All data sets were processed using XDS (Kabsch, 2010)with the xdsme script (Legrand, P. XDSME: XDS Made Easier (GitHub Repository, 2017); https://github.com/legrandp/xdsme and https://doi.org/10.5281/zenodo.837885), before being scaled together with XSCALE and run through XDSME/XDSCONV to generate MTZ files. The structure was solved by molecular replacement using Phaser (McCoy et al., 2007), starting from PDB 1BIK as reference model, followed by refinement using REFMAC5 (Murshudov et al., 2011). The electron density maps were viewed in COOT (Emsley et al., 2010). The structures were subjected to at least three cycles of rebuilding and refinement performed by using REFMAC5 (Murshudov et al., 2011), phenix.refine (Murshudov et al., 2011) and BUSTER (Smart et al., 2012). The figures were made with PyMOL (Mooers and Brown, 2021). The structure was deposited into the Protein Data Bank (PDB): 8AJ7 (Chudzinski-Tavassi et al., 2020).

### Compounds preparation

Synthetic peptides provided by CEA were solubilized in H_2_O. The homogeneous recombinant protein Amblyomin-X was prepared according to standard protocols previously reported by our group (Batista et al., 2010; Morais et al., 2016; Maria et al., 2019).

### Cell lines and culture conditions

Human’s melanomas (SK-MEL-28) and pancreatic adenocarcinomas (MIA PaCa-2) cells were obtained and cultured according to instructions of American type culture collection (ATCC, Manassas, VA). All cell types were routinely grown in a humidified 5% CO_2_ incubator at 37 °C.

### Cell viability assay

Cell viability was measured by 3-(4,5-dimethylthiazol-2-yl)-2,5-diphenyltetrazolium bromide (MTT) assay, as described elsewhere (Morais et al., 2016). Briefly, SK-MEL-28 and MIA PaCa-2 cells were seeded in 96-well plates (10^4^ cells/well) and incubated with: (i) C-ter and Kunitz synthetic domains in the concentrations range: 0.2–100 μM; (ii) p15, a protein Kinase (casein kinase 2, CK2) inhibitor that shows the antitumor effect when fused to the cell-penetrating peptide derived from the HIV-TAT protein, or this molecule conjugate to C-ter (p15-C-ter), both at 10 and 100 μM; (iii) TAT-Kunitz at 20 and 50 μM. After 48h, 10% of 5 mg/ml MTT was added to the cells and the plates were incubated for 3 h at 37 °C. Afterward, the medium was discarded, the dark blue formazan crystalline product was dissolved in 100 µL dimethyl sulfoxide (DMSO), and the absorbance was measured in a Spectra MAX 190 microplate reader (Molecular Devices, Sunnyvale, United States) at 540 nm.

Amblyomin-X (50 μM) were used for comparative purpose. Besides, staurosporine (5 μM), MG-132 (3 μM) and bortezomib (80 nM) were used as a positive control.

### Cellular uptake studies

For detection, full Amblyomin-X and synthetic peptides (C-ter, C-ter-p15, p15, Kunitz, TAT-Kunitz) were directly labeled with non-cell-permeable amine-reactive dye (Alexa Fluor 488 carboxylic acid tetrafluorophenyl (TFP) ester) using the Microscale Alexa Fluor^®^ 488 Protein Labeling kit (Molecular Probes), following manufacturer instruction. An additional step was carried out for the purification of peptides using FPLC. SK-MEL-28 and MIA PaCa-2 (10^5^ cells/well) were seeded on sterile 35 mm culture dishes. First, a cell-based assay using fluorochrome conjugated molecules was carried by incubation with Amblyomin-X and its synthetic domains (20 µM) in a fresh medium at 37°C for 4 h and nuclei were stained with 5 mM Syto59 (Invitrogen Life Technologies Inc., United States). Then red and green fluorescence were monitored by LSM 510 Meta confocal microscope (Zeiss, Jena, Germany). Confocal microscopy analysis were also applied for investigation of C-ter derived fragments synthesized as FITC-conjugate and named F1C, F2C and F3C. After treatment with synthetic fragments (all at 5 µM) in a fresh medium at 37°C for 4h, tumor cells were scanned on the x-, y-, and *z*-axes using a confocal microscope TCS SP8 (Leica Microsystems, GmbH Mannheim, DE) with 40x/1.1 objective lens and laser excitation at 405 and 488 nm with additional brightfield channel using the LAS X software (Leica Microsystems, GmbH Mannheim, DE). The images were deconvolved in Huygens Essential version 22.04 (Scientific Volume Imaging, Netherlands).

Additionally, tumor cells were seeded on 96 wells plates were incubated with recombinant Amblyomin-X or synthetic C-ter and Kunitz (all at 20 µM) at 37°C for different times of treatment. Following the same rationale, after treatment, cells were incubated with Alexa Fluor 555 TrF (100 μg/ml), Alexa Fluor 555 CTxB (50 μg/ml) or Alexa Fluor 555 dextran (1 mg/ml) in the binding medium at 37 °C for 20 min. Also, to evaluate cargo-delivery ability of C-ter, tumor cells were incubated with p15 (100 μM) or this molecule conjugate to C-ter (100 μM) for 4 h. Similarly, enhancement of Kunitz uptake by using CPP delivery tool was evaluated by treatment with Kunitz (50 μM) and TAT-Kunitz (50 μM) for 4 h. Finally, evaluation of C-ter derived fragments (all at 10 μM) were investigated after 4 h of treatment.

In all 96 wells plate assays, nuclei were stained with Hoechst 33342 and fluorescence was monitored by a Molecular Devices ImageXpress Micro Confocal plate-scanning microscope (Molecular Devices, Sunnyvale, CA). Vesicles count were analyzed using MetaXpress software (Molecular Devices, CA) with Transfluor Application Module.

### Microinjection assay

SK-MEL-28 cells were plated on glass Cellocate coverslips for 24 h before injection. The microinjection solution was prepared using fluorochrome conjugated molecules: 488-Amblyomin-X (20 µM) or 488-Kunitz (10 µM). Staurosporine (10 µM) was added directly to the medium for 6 h and was used as a control for PI staining. Ctrl cells were maintained in a culture medium. The solution was injected into the cytoplasm an average of 50 cells for each condition (compensation pressure: 30 hPa; injection pressure: 100 hPa; injection time: 0.3 s). Immediately after injection, cells were replaced with fresh medium in the incubator at 37 °C. After 4 h, cells were stained with PI (1 μg/ml – contain RNAse A 100 ng/ml) for 15 min at r.t.. Then, cells were washed twice with 1X PBS and fixed with 4% paraformaldehyde for 15 min at r.t... Finally, cells were washed with 1X PBS and one drop of solution anti-fade mounting Vectashield^®^ (Vecta Labs) was applied on the slide with the coverslip containing the cells facing down and, then, sealed. The analysis was performed in Leica SP8 confocal microscope (Leica, Germany): Green fluorescence was used to identify microinjected cells and red fluorescence to score cell death.

### Intracellular trafficking by indirect Immunofluorescence

Tumor cells were grown on 96-well plates (10^4^ cells/well) and treated with 488-C-ter (20 µM) for indicated times. Cells were washed twice with PHEM-glycine buffer (2 mM HEPES, 10 mM EGTA, 2 mM MgCl_2_, 60 mM Pipes, 100 mM glycine, pH 6.9) and fixed with 4% paraformaldehyde for 3 h at room temperature. The washing step was repeated and, then, cells were incubated with a cell permeabilization solution (0.1% Tween in PHEM) for 5 min at r.t. Samples were washed and were incubated with blocking solution 1% BSA for 30 min at r.t.. Following, the primary antibody was incubated overnight at 4 °C: (i) anti-human caveolin-1 mouse 1:100 (Santa Cruz Biotechnology, Inc., United States); (ii) anti-human LAMP-2 rabbit 1:50 (Abcam). A washing procedure was carried out and samples were incubated with secondary antibodies Alexa Fluor^®^ 555 goat anti-rabbit and Alexa Fluor ^®^ 647 rabbit anti-mouse (Invitrogen™ Life Technologies Inc., United States), both at 1:200 dilution for 1 h at r.t. in the dark. Nuclei were stained with Hoechst 33342. Then, green and red fluorescences were monitored by a Molecular Devices ImageXpress Micro Confocal plate-scanning microscope (Molecular Devices, Sunnyvale, CA) followed by an evaluation of overlap between 488-C-ter and endocytosis markers using MetaXpress software (Molecular Devices, Sunnyvale, CA).

### Gene expression

Gene expression was evaluated *via* quantitative real-time polymerase chain reaction (qPCR) according to standard protocols previously reported by our group (Pacheco et al., 2014; Morais et al., 2016). Briefly, after cell treatment, the total RNA was extracted using the RNeasy kit (Qiagen, Netherlands) followed by DNase treatment, Then, cDNA strands were constructed using a SuperScript^®^ III First-Strand Synthesis kit (InvitrogenTM, Life Technologies Inc., United States), according to manufacturer’s instructions. Finally, the samples were applied in a SYBR^®^ green (Applied Biosystems, United States)-based reaction with specific and validated primers to evaluate mRNA levels of genes coding for dynein chains. RNA levels were normalized to glyceraldehyde 3-phosphate dehydrogenase (GAPDH) and quantified using the Livak 2^−ΔΔCT^ method (Livak and Schmittgen, 2001). The experiments were conducted by Step One Plus^®^ PCR real-time system (Applied Biosystems, United States).

## Statistical analysis

Data are expressed as mean ± SD of three independent experiments. Comparisons were made using GraphPad Prism 5.0 software (GraphPad Software Inc., San Diego, CA). For cell viability assay, statistical significance was determined by *t*-test or one-way analysis of variance (ANOVA) followed by the Dunnett post-test. Statistical analyses for nuclear translocation and overlap between C-ter-488 and endocytosis markers were performed comparing each time of treatment to identify the more relevant by two-way ANOVA with Bonferroni post-test. For vesicle counts, statistical significance was determined by *t*-test considering non-conjugated molecules as reference. The criteria for statistical significance were set up as *p ≤ 0.05, **p ≤ 0.01 and ***p ≤ 0.001.

## Data Availability

The datasets presented in this study can be found in online repositories. The names of the repository/repositories and accession number(s) can be found below: http://www.wwpdb.org/, 8AJ7.
