# Supplementary figures and images for: Structural and functional properties of the Kunitz-type and C-terminal domains of Amblyomin-X supporting its antitumor activity
Source: Front Mol Biosci. 2023 Feb 9;10:1072751. doi: 10.3389/fmolb.2023.1072751 (PMC9948614; doi:10.3389/fmolb.2023.1072751)

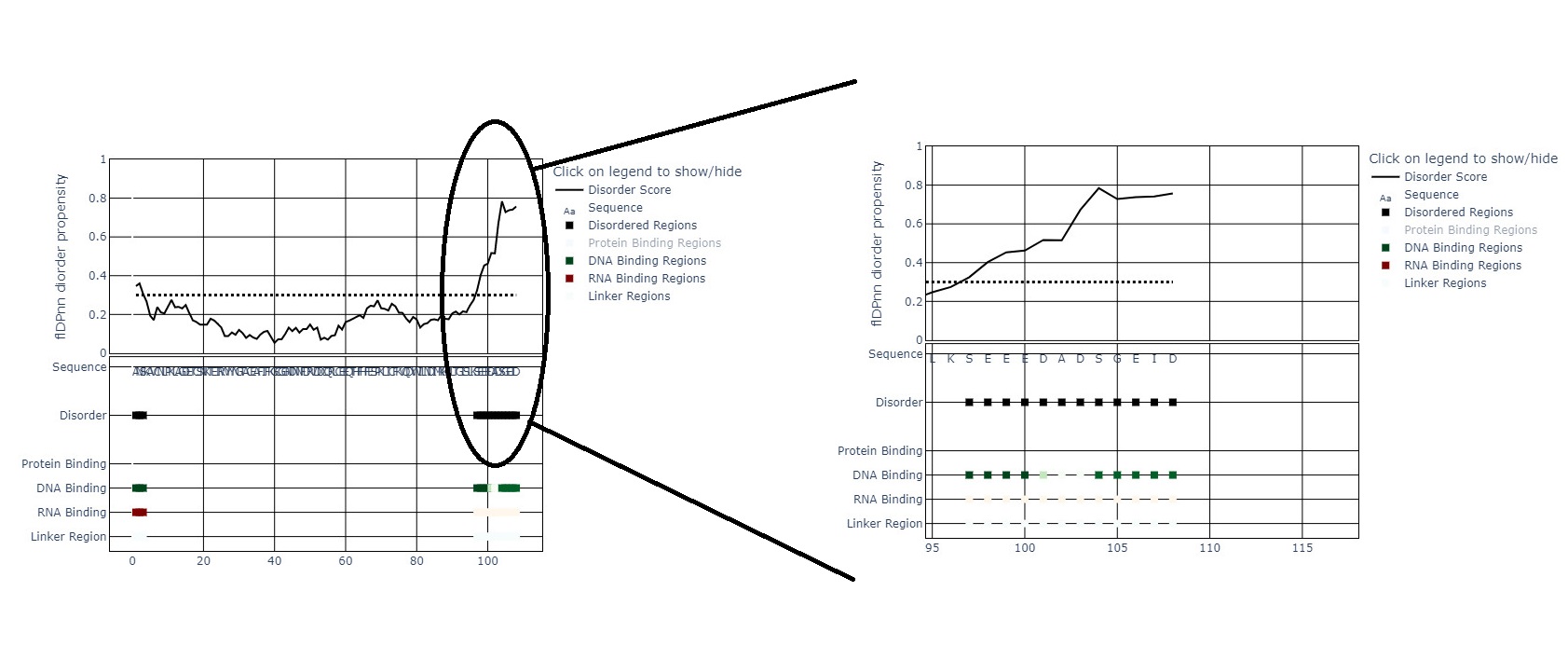

Supplement: Supplementary file 1 [file Image3.JPEG]

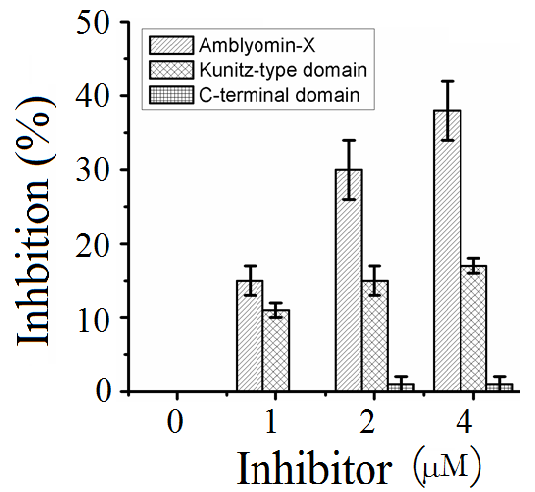

Supplement: Supplementary file 2 [file Image4.TIF]

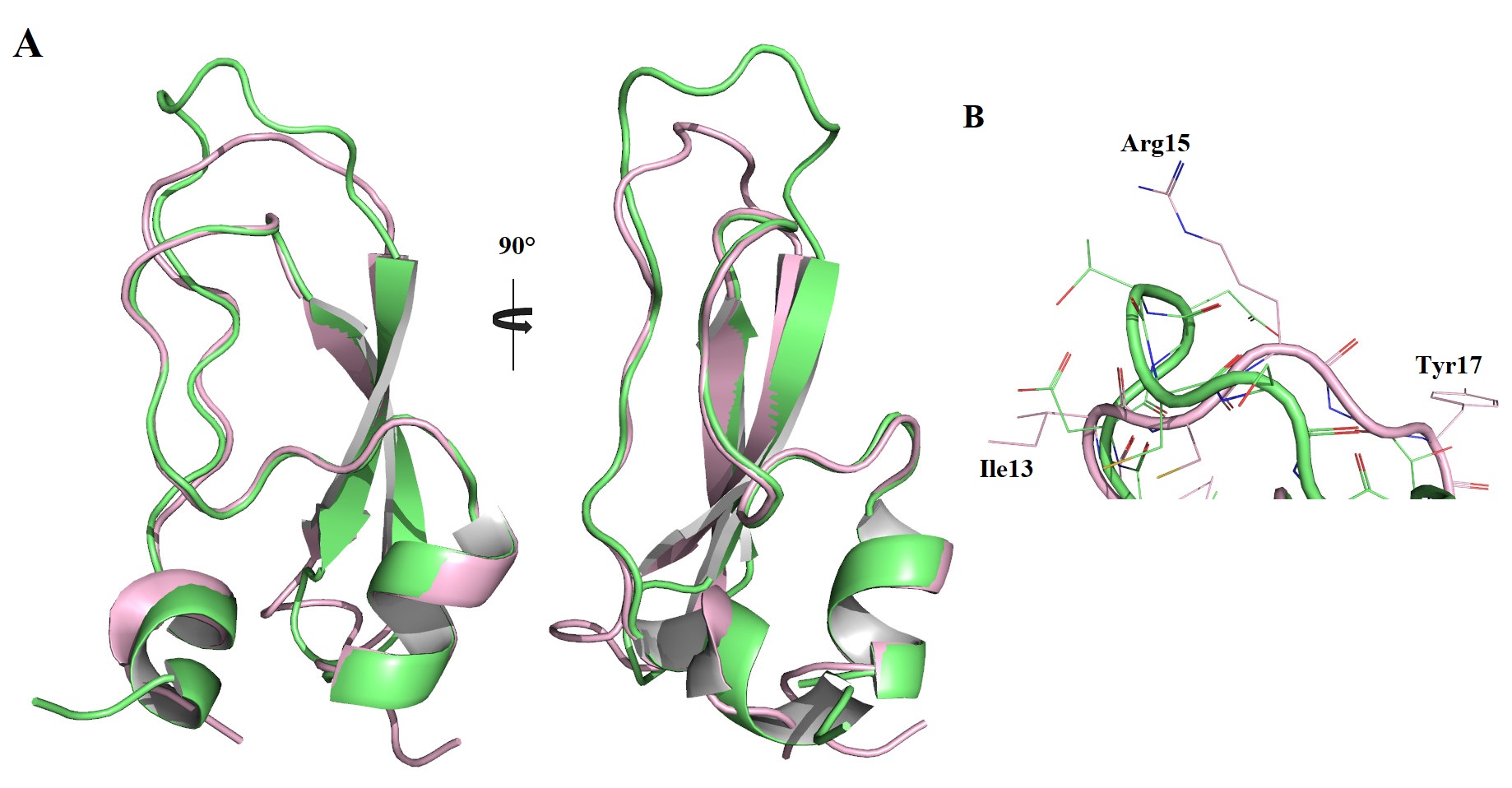

Supplement: Supplementary file 3 [file Image2.JPEG]

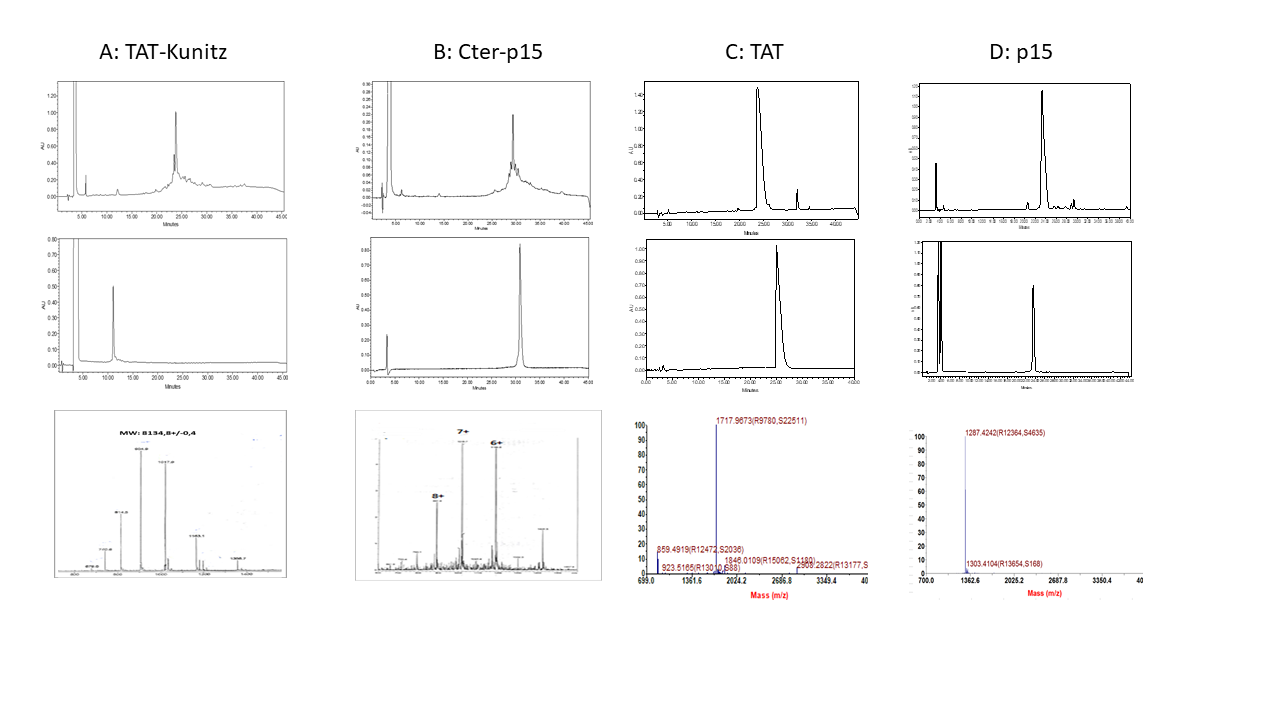

Supplement: Supplementary file 4 [file Image1.TIF]

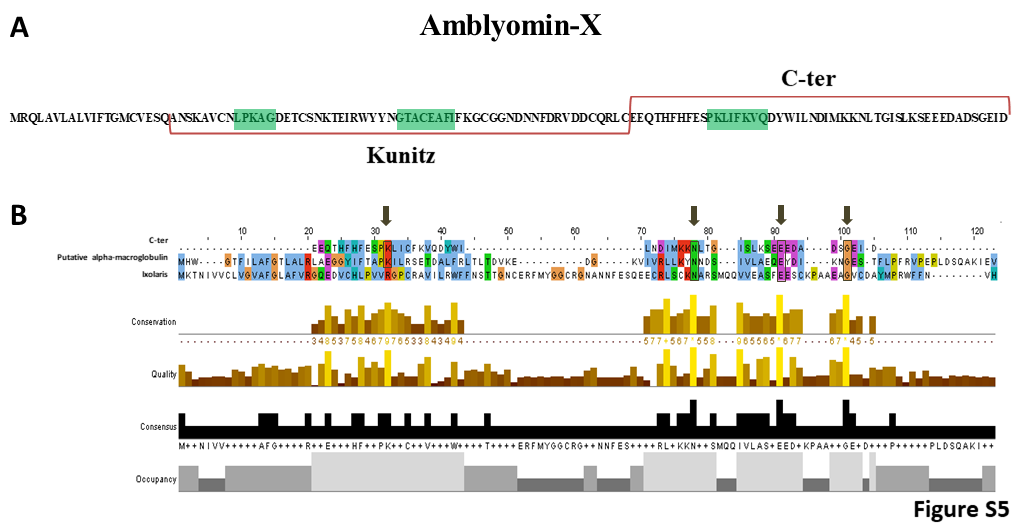

Supplement: Supplementary file 5 [file Image5.TIF]
