# Supplementary material for: Structural and functional properties of the Kunitz-type and C-terminal domains of Amblyomin-X supporting its antitumor activity
Source: Front Mol Biosci. 2023 Feb 9;10:1072751. doi: 10.3389/fmolb.2023.1072751 (PMC9948614; doi:10.3389/fmolb.2023.1072751)
Supplement: Supplementary file 6 [file DataSheet1.docx]

**SUPPLEMENTAL MATERIAL**

**Supplementary material 1 - FXa activity assays -** FXa (40 nM) was incubated with varying concentrations of the Amblyomin-X recombinant protein and the synthetic domains for 30 min at 37 °C in 20 mM Tris–HCl containing PS/PC (2 µM) and 5 mM CaCl2, pH 8.0. Cleavage of the chromogenic substrate S-2765 (200µM, Chromogenix, UK) was monitored at 405 nm (SpectraMax 190, Molecular Devices, USA). The percentage of inhibition was determined from the residual activity of the enzymes as previously reported15, 16.

**Supplementary material 2 - The alignment of multiple protein sequences -** C-ter amino acid sequence was aligned with selected sequences by Jalview 2.10.5 using Clustal O method and the default parameters.

**Supplementary material 3 -** **Identification of fragments of Amblyomin-X domains in cell lysate through mass spectrometry analysis.** Lysates were prepared from tumor cells treated or untreated with Amblyomin-X. Then, samples were injected on HPLC on C18 column (Supelco, 3 μM, 100 Å, 50 mm x 2.1 mm). The gradient used was 0 to 40% B (90% acetonitrile in water containing 0.1% formic acid) in 20 min in a constant flow of 0.2 mL/min. The eluate content was analyzed by mass spectrometry, in positive mode, with interface voltage parameters at 4.5 kV, detector voltage at 1.76 kV, and interface temperature at 200°C. Analysis in MS mode was done with the acquisition of data in the range of 50 to 2000 m/z, and for MS/MS mode analysis, automatic ion fragmentation was performed according to the relative intensity caused by collision by argon gas, with 50% energy. MS/MS spectra were also obtained in the range of 50 to 2000 m/z. The results were analyzed using the MASCOT online platform (http://www.matrixscience.com) through the MS/MS ion search in a NCBI database. The parameters used were: carbamidomethyl (C) as fixed modification; oxidation (M) as variable modification; peptide tolerance ± 0.5 Da; peptide charge +1, +2 and +3.

**Figure S1. HPLC chromatograms.** HPLC chromatograms of the crude (first line) and refolded and/or purified (second line) peptides and Mass spectrum of the purified peptides (third line): TAT-Kunitz (A), C-ter-p15 (B), TAT ©, and p15(D)e.

**Figure S2. Comparison of Kunitz domain structures.** A. Superimposition between Kunitz-domain of Amblyomin-X (green) and TFPI-K2 (pdb 1TFX, pink). B. Zoom of the loop that interacts with FXa

**Figure S3.** Graphic representation of predicted disordered regions calculated by flDnn server **(**[**http://biomine.cs.vcu.edu/servers/flDPnn/**](http://biomine.cs.vcu.edu/servers/flDPnn/)**)**

**Figure S4. FXa inhibition by Amblyomin-X depends on the full protein, but not the isolated domains.** The hydrolysis of chromogenic substrate by FXa (40 nM) in 20 mM tris–HCl containing PS/PC (2 uM) and 5 mM CaCl2, pH 8.0 was monitored (in the absence or presence of increasing concentration of inhibitors) spectrophotometrically at 405 nm.

**Figure S5:** **Fragments of Amblyomin-X domains Amblyomin-X in tumor cell lysate and** **comparison between C-ter and other proteins.** (A) The entire sequence of Amblyomin-X and the delimitation of Kunitz and C-ter domains. Highlighted in green, fragments of both domains found in lysate generated from tumor cells treated with Amblyomin-X through mass spectrometry analysis (B) Multiple alignments of the C-terminal (C-ter) domain of Amblyomin-X and other proteins using ClustalW2 and Jalview. Ixolaris (UniProtKB: B7P3L9) and Putative alpha-macroglobulin (UniProtKB: A0A1E1XVY5). The arrows indicate the specific residues conserved between C-ter and other molecules. The amino acid residues were colored by percentage identity. Conservation of sequences can be seen as bars, scored as 9 to 10 (or *), meaning fully conserved.
